# Exophthalmic Goitre

**Published:** 1902-11-08

**Authors:** 


### EXOPHTHALMIC GOITRE.

The drift of the discussion on exophthalmic goitre which took place at the last meeting of the Royal Medical and Chirurgical Society, was to suggest a greater hopefulness in the prognosis of this disorder than some have been willing to admit. Dr. George R. Murray, of Newcastle, related the results of his observation of 120 cases, of which 110 had occurred in women and only 10 in men. The onset of the disease being nearly always insidious the age of onset was difficult to determine, but the disease had most frequently started in the earlier decades of life, the greatest liability to it being between 15 and 35 years of age. In several cases brothers or sisters of the patients had suffered from the same malady, but there was no history of goitre in either parent of any case. As to antecedent conditions, it appeared that sudden and prolonged grief, or events producing powerful emotions, had been the exciting causes in some of the cases. The first symptom noted was generally the goitre, but in 19 cases it was palpitation, while in three of the cases there was no thyroid enlargement. In all the cases the pulse rate was increased, ranging from 90 up to 200 per minute; in 66 cases it was between 120 and 150, but there was great variation in individual cases from time to time. In 33 of the cases no exophthalmos was present, and in three, such as had existed disappeared. The course of the disease was variable. In one case it lasted only nine months, and ended in recovery; in another death occurred within a year. In a considerable number of cases practical recovery took place, although usually some relic of the condition persisted. In no case did myxœdema ensue. As to treatment thyroid gland was harmful, but thymus and supra-renal extract had proved useful. In severe cases rest in bed for three or four weeks was essential, and if there were much wasting Weir-Mitchell treatment was indicated. For less severe cases a quiet life with much time spent in the open air was best. Gentle Faradism for an hour night and morning was useful, and should be carried on for several months. Convallaria had proved more effectual than other cardiac tonics in lowering the pulse rate. Bromides were useful for the accompanying nervousness, and arsenic in moderate doses gave good results in almost all cases.

Dr. Hale White pointed out that there was some relation between this disease and rheumatic tendencies, and that there was also a close association with mental disease. As to treatment, belladonna was rarely if ever useful, neither were thymus or supra-renal tablets. The best treatment was probably hygienic with quietness.

Dr. Ewart thought that the disease depended upon a chronic intoxication in susceptible subjects, the diarrhœa which was so often seen, although partly paralytic, was probably often toxic or fermentive, while even the thyroid changes were probably the result of a toxic influence.

In reference to the question of fright or anxiety being causes of the disease Dr. Rolleston mentioned the increased prevalence of the cases since the war, and described the case of a yeoman who developed symptoms after serving in South Africa. He thought the prognosis was not very bad, as fatal cases were in his experience rare.

